# Can we optimise doxorubicin treatment regimens for children with cancer? Pharmacokinetic simulations and a Delphi consensus procedure

**DOI:** 10.1186/s40360-020-00417-2

**Published:** 2020-05-28

**Authors:** Christian Siebel, Gudrun Würthwein, Claudia Lanvers-Kaminsky, Nicolas André, Frank Berthold, Ilaria Castelli, Pascal Chastagner, François Doz, Martin English, Gabriele Escherich, Michael C. Frühwald, Norbert Graf, Andreas H. Groll, Antonio Ruggiero, Georg Hempel, Joachim Boos

**Affiliations:** 1 Department of Paediatric Haematology and Oncology, University Children’s Hospital Muenster, Albert-Schweitzer-Campus 1, A1, 48149 Muenster, Germany; 2 Department of Paediatric Haematology-Oncology, La Timone University Hospital of Marseille, Marseille, France; 3 Department of Paediatric Oncology and Haematology, University Children’s Hospital Cologne, Cologne, Germany; 4 Department of Paediatrics, University of Milano-Bicocca, Hospital S Gerardo, Monza, Italy; 5 Department of Paediatric Oncology, CHRU Nancy, Vandoeuvre Les Nancy, France; 6 Oncology Center SIREDO, Institut Curie and University Paris Descartes, Paris, France; 7 Birmingham Women’s and Children’s Hospital NHS Foundation Trust, Birmingham, UK; 8 University Medical Centre Eppendorf, Clinic of Paediatric Haematology and Oncology, Hamburg, Germany; 9 Swabian Children’s Cancer Centre, University Children’s Hospital Augsburg, Augsburg, Germany; 10 Department of Paediatric Haematology/Oncology, Saarland University, Homburg/Saar, Germany; 11 Division of Paediatric Oncology, Catholic University of Rome, Rome, Italy; 12 Department of Pharmaceutical and Medical Chemistry - Clinical Pharmacy, University of Muenster, Muenster, Germany

**Keywords:** Doxorubicin, Children, Cardiotoxicity, Pharmacokinetics, Delphi procedure

## Abstract

**Background:**

Despite its cardiotoxicity doxorubicin is widely used for the treatment of paediatric malignancies. Current treatment regimens appear to be suboptimal as treatment strategies vary and do not follow a clear pharmacological rationale. Standardisation of dosing strategies in particular for infants and younger children is required but is hampered by scarcely defined exposure-response relationships. The aim is to provide a rational dosing concept allowing for a reduction of variability in systemic therapy intensity and subsequently unforeseen side effects.

**Methods:**

Doxorubicin plasma concentrations in paediatric cancer patients were simulated for different treatment schedules using a population pharmacokinetic model which considers age-dependent differences in doxorubicin clearance. Overall drug exposure and peak concentrations were assessed. Simulation results were used to support a three round Delphi consensus procedure with the aim to clarify the pharmacological goals of doxorubicin dosing in young children. A group of 28 experts representing paediatric trial groups and clinical centres were invited to participate in this process.

**Results:**

Pharmacokinetic simulations illustrated the substantial differences in therapy intensity associated with current dosing strategies. Consensus among the panel members was obtained on a standardised a priori dose adaptation that individualises doxorubicin doses based on age and body surface area targeting uniform drug exposure across children treated with the same protocol. Further, a reduction of peak concentrations in very young children by prolonged infusion was recommended.

**Conclusions:**

An approach to standardise current dose modification schemes in young children is proposed. The consented concept takes individual pharmacokinetic characteristics into account and involves adaptation of both the dose and the infusion duration potentially improving the safety of doxorubicin administration.

## Background

Since their introduction to chemotherapy in the 1960s anthracyclines have gained widespread use in the treatment of solid and haematological malignancies. Today, roughly 60% of children with cancer receive anthracyclines, most commonly doxorubicin (DOX). Anthracyclines significantly increase event-free survival in Ewing’s sarcoma and a better antitumor efficacy is suggested for acute lymphoblastic leukaemia (ALL) [1]. However, a well-known drawback of this class of cytostatics is the induction of progressive chronic cardiotoxicity which is associated with cardiomyopathy, congestive heart failure and elevated mortality [2]. The risk of irreversible cardiac damage has been associated in particular with the cumulative lifetime anthracycline dose and age which exposes the youngest patients to the highest risk [3–5].

Much attention has been paid to measures seeking to prevent adverse cardiac effects, which, inter alia, include cardioprotective agents such as dexrazoxane as well as liposomal anthracyclines [6, 7]. Administration of weekly split-doses rather than one large dose as well as prolonged continuous infusions were among the most studied strategies. Essentially, these approaches rely on the assumption that drug exposure (expressed as area under the concentration-time curve (AUC)) is the most important determinant of antitumor efficacy and that reduced peak concentrations (c_max_) mitigate the toxic cardiac effect of anthracyclines without affecting their antitumor activity [8]. After 50 years of clinical use a plethora of studies in adult patients have been performed supporting the rationale of prolonged continuous infusion or split-dose schedules to reduce cardiotoxicity [8–10]. For paediatrics, however, the situation is less clear due to the paucity of well-designed randomized trials [11–14]. Our understanding of the consequences of pharmacokinetic (PK) parameters such as AUC and c_max_ for both toxicity and efficacy in paediatrics still remain insufficient. It has been suggested that the positive effect of continuous infusion seen in adult patients is attributed to a reduction of the cardiac anthracycline concentration [8]. However, one might argue that in children with a developing heart, longer exposure due to prolonged infusion might be just as toxic as high peak concentrations.

Underscoring the high demand on more information on the PK and safety of DOX in paediatrics, the European Medicines Agency put DOX on their 2007 ‘priority list on studies for off-patent medicinal products’ supporting the conduct of further trials (doc. Ref. EMEA/197972/2007, London, June 2007). Based on data from the EPOC-MS-001-Doxo trial Völler et al. demonstrated that the DOX clearance normalized to body surface area (BSA) is significantly lower in children below the age of 3 years compared to older children [15]. Besides age and BSA, other covariates such as genetic polymorphisms in genes responsible for drug transport and metabolism or the tumour entity did not have an effect on DOX PK. Though the physiological basis is unclear, the results of the EPOC trial raise the question whether the reduction of clearance and its effect on individual systemic therapy intensity is of direct clinical importance and should impact dosing recommendations.

When looking at current treatment regimens in paediatrics, one is faced with a multitude of DOX doses, infusion times and instructions for dose modifications (see Additional file 1 for a selection of current protocols) [16, 17]. Protocols for children have evolved over time and are rather based on empirical grounds than following a sound pharmacological rationale. This becomes particularly apparent with the large variability of dose modification schemes that are used for the youngest children. Dose modifications applied to infants and young children below a certain age or body weight are justified by the higher risk of late cardiac abnormalities in this patient group, yet age- and/or body weight-based boundaries and conversion rules from BSA-based dosing to body weight-based dosing are seemingly arbitrary. Exemplarily, the CWS-SoTiSaR guidance recommends switching from BSA-based dosing to body weight-based dosing in children below 1 year or weighing less than 10 kg (see Additional file 1). A further dose reduction to 67% of the body weight-based dose is intended for children below 6 months. In contrast, according to the NHL-BFM 2012 registry children below the age of 1 year should receive 75% of the BSA-based dose and children below 6 months 67% of the BSA-based dose. Obviously, these dose modifications result in large discrepancies of the DOX dose across children of different age but treated for the same cancer. Furthermore, as soon as a child reaches a particular age−/body weight boundary there will be a sudden increase in the DOX dose.

Undoubtedly, with increasing numbers of childhood cancer survivors the prevention of cardiotoxic late effects must be given top priority. This also implies that the current practice of DOX administration needs to be critically questioned. Both factors described here, age-dependent differences of the DOX clearance as well as empirical dose modifications will affect the therapy intensity experienced by the individual child with unknown influence on therapy efficacy and safety. A standardised dosing strategy for young children which adequately reflects individual PK characteristics would therefore be highly desirable. As pointed out by Völler et al. the DOX population PK model provides a tool to develop more rationale alternative dosing strategies [16]. However, as described in the beginning a well-defined target PK parameter on which to base such a dosing strategy is still lacking. In this paper we will propose an approach how standardising dosing strategies for young children could be achieved. At first, we will visualise the influence of empirical dose modifications and age-dependent PK differences on therapy intensity (AUC and c_max_) for a number of selected treatment protocols thereby demonstrating the shortcomings of current approaches. Secondly, we will clarify the goals of future dosing recommendations in young children. For the latter, a Delphi consensus procedure among expert paediatric oncologists was conducted. In perspective, the approach described here will provide the possibility to validate a common dosing strategy in young children across the different study groups taking into account the specific dose intensity for each tumour entity.

## Methods

### Pharmacokinetic simulations

To visualize the impact of current dosing recommendations along with age-dependent differences in PK on drug exposure and peak concentrations Monte Carlo simulations were carried out using a population PK model published by Völler et al. [15]. The model was built upon PK data from 94 patients from the EPOC-MS-001-Doxo trial (see Additional file 3 for a short description of the patients). This patient cohort was considered to represent typical paediatric cancer patients. Simulations of children aged 0–18 years with demographics taken from WHO and CDC growth charts were performed and DOX doses and infusion times from a selection of currently applied paediatric treatment regimens were analysed (see Additional file 1) [18]. Individuals on the 5th, 50th or 95th percentile of body height and weight were simulated. Model parameters were fixed for simulations to the final parameter estimates of the EPOC patient population. To display the typical course of AUC and c_max_ for a median child inter-individual and intra-individual variability were set to zero. Simulations including inter- and intra-individual variability were performed to display the remaining variability that cannot be explained by age and BSA. This variability represents the uncertainty associated with any model-based prediction. Each individual was simulated 1000 times.

In order to illustrate the effect of a standardized model-based dose calculation rule on drug exposure, observed AUC values for 94 patients from the EPOC cohort were compared with hypothetical, dose-adjusted AUC values. Calculation of dose-adjusted AUC values was based on a dose adaptation previously described by Völler et al. [16]. This dose adaptation takes individual age and BSA into account, as these parameters were identified as predictive covariates for DOX PK [15]. As reference AUC value for the suggested dose adaptation the AUC of an 18-year-old boy was chosen. For the purpose of this paper, we will refer to this reference value as ‘target AUC’. Based on the model-predicted clearance CL_18 years_ for a typical 18-year-old boy with median demographics (eq. 1), this target AUC_18 years_ was determined as 344 μg·L^− 1^·h for a reference dose of 10 mg·m^− 2^ (1278 μg·L^− 1^·h for a reference dose of 1 mg·kg^− 1^ in case of body weight-based dosing). An adjusted DOX dose was obtained for each patient from the EPOC cohort according to eqs. 1 and 2. Firstly, the model-predicted clearance was estimated considering each patient’s age and BSA (eq. 1). Secondly, the adjusted DOX dose was calculated (eq. 2), where Dose_18_ is the absolute DOX dose for the typical 18-year-old boy specified by the respective treatment regimen.

1 $$ {CL}_{model- predicted}=9.26\ast \left(1+\left( BSA-0.77\right)\ast 1.30\right)\ast \left(1+{\left(\frac{AGE}{5.32}\right)}^{0.286}\right) $$

2 $$ {Dose}_{ind}={Dose}_{18\  years}\ast \frac{CL_{model- predicted}}{CL_{18\  years}} $$

Based on the observed and adjusted DOX doses, observed and dose-adjusted AUC values were calculated according to eq. 3 with CL_EPOC_ denominating the empirical Bayesian clearance estimates derived from the EPOC-MS-001-Doxo data.

3 $$ AUC= Dose/{CL}_{EPOC} $$

To allow comparison across different treatment regimens and to illustrate deviations from the target, observed and dose-adjusted AUC values were normalised to the regimen-specific target AUC. Bias and precision were calculated for both groups as median prediction error and median absolute prediction error according to Sheiner and Beal [19]. The probability to attain a target range of 80–125% was calculated for both groups. The range of 80–125% around the target AUC was adopted from bioequivalence standards [20].

### Data and statistical analysis

Monte Carlo simulations were carried out in NONMEM® version 7.3 [21]. R version 3.5.0 [22] and RStudio version 1.1.456 [23] were used for graphical representation of simulation results and statistical analysis. Non-parametric Wilcoxon signed rank test was performed on continuous data and McNemar’s chi-squared test was performed on paired nominal data. A *p* value < 0.05 was deemed statistically noticeable. Confidence intervals for the median were calculated using the function ‘quantileCI’ provided by the R package ‘jmuOutlier’ which calculates exact confidence intervals on quantiles based on the binomial test.

### Delphi consensus procedure

Overall 28 paediatric oncologists were invited to participate in a three round Delphi consensus procedure. While the main focus of the Delphi procedure was to establish a consensus on the goals of hitherto protocol-specific DOX dose modifications in young children, a couple of further questions were asked covering general goals of DOX administration as well as additional aspects that were raised by the panel members during the first round of the Delphi process. A detailed description of the methodology can be found in Additional file 4.

## Results

### Visualising the therapy intensity achieved with current protocols

**Fig. 1:**
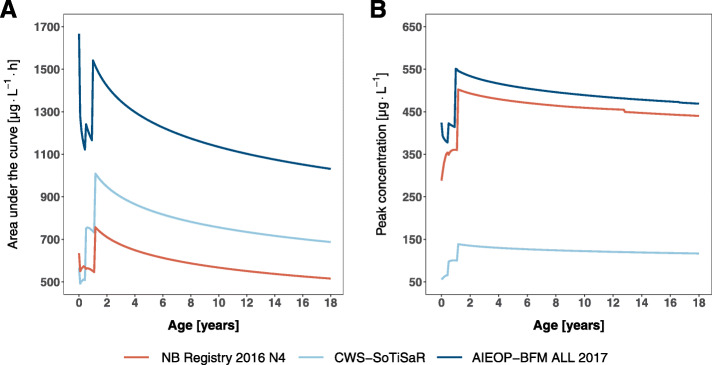
DOX AUC (**a**) and c_max_ (**b**) across the age range from 0 to 18 years. Typical AUC and c_max_ values were simulated for children on the 50th percentile of body height and weight for three selected treatment regimens. Underlying DOX doses were adjusted as specified by the respective regimen. NB Registry 2016 N4 (standard dose: 15 mg·m^− 2^, 0.5 h): reduction to 100% of the body weight-based dose in children < 12 months or < 10 kg; CWS-SoTiSaR (20 mg·m^− 2^, 3 h): reduction to 67% of the body weight-based dose in children < 6 months and reduction to 100% of the body weight-based dose in children ≥6 months but ≤10 kg; AIEOP-BFM ALL 2017 (30 mg·m^− 2^, 1 h): reduction to 67% of the BSA-based dose in children < 6 months and reduction to 75% of the BSA-based dose in children 6–12 months. The grey boxes mark the areas of the curve where doses were reduced. For a child on the 50th percentile of body height and weight the threshold for dose reduction is reached at an age of 14 months exceeding a body weight of 10 kg (NB Registry 2016 N4, CWS-SoTiSaR) or 12 months (AIEOP-BFM ALL 2017), respectively

We performed simulations for three selected treatment protocols to illustrate the discrepancies in systemic therapy intensity achieved with current treatment protocols. For each protocol the simulations visualise the joint impact of current dose modification schemes and age-dependent PK differences on AUC and c_max_. As outlined by Fig. 1 exemplarily for three treatment regimens, substantial differences in drug exposure and peak concentrations have to be expected across children aged 0–18. In very young children who are subject to dose reductions these may lead to particularly sharp steps in therapy intensity. To illustrate this in more detail we will use the example of a child treated according to the CWS-SoTiSaR guidance (dose: 20 mg·m^− 2^, infusion time: 3 h). Here, the standard BSA-based dose is reduced in children < 6 months to 67% of the body weight-based dose and in children 6–12 months or < 10 kg to 100% of the body weight-based dose (see Additional file 1). As the ratio of body weight to BSA is lower in infants than in older children moving from BSA- to body weight-based dosing leads to a reduction of the administered DOX dose. In this case, simulated typical AUC and c_max_ are lowest in neonates (AUC = 507 μg·L^− 1^·h, c_max_ = 56 μg·L^− 1^) and increase towards a maximum in children slightly above 1 year of age (AUC = 1002 μg·L^− 1^·h, c_max_ = 138 μg·L^− 1^) which highlights the impact of the dose modification. Despite the decrease in clearance in young children, using this dose reduction scheme the lowest therapy intensity is experienced by the youngest children. Of note, typical AUC and c_max_ for children 2 months and younger are extrapolated as the youngest child included in the EPOC-MS-001-Doxo trial was 2.5 months old. Due to the increase in DOX clearance with growing age, simulated typical AUC decreases from its maximum 1002 μg·L^− 1^·h in a child slightly above 1 year of age to 688 μg·L^− 1^·h at the age of 18. Similarly, however less pronounced, typical c_max_ decreases from 138 μg·L^− 1^ to 117 μg·L^− 1^.

**Fig. 2:**
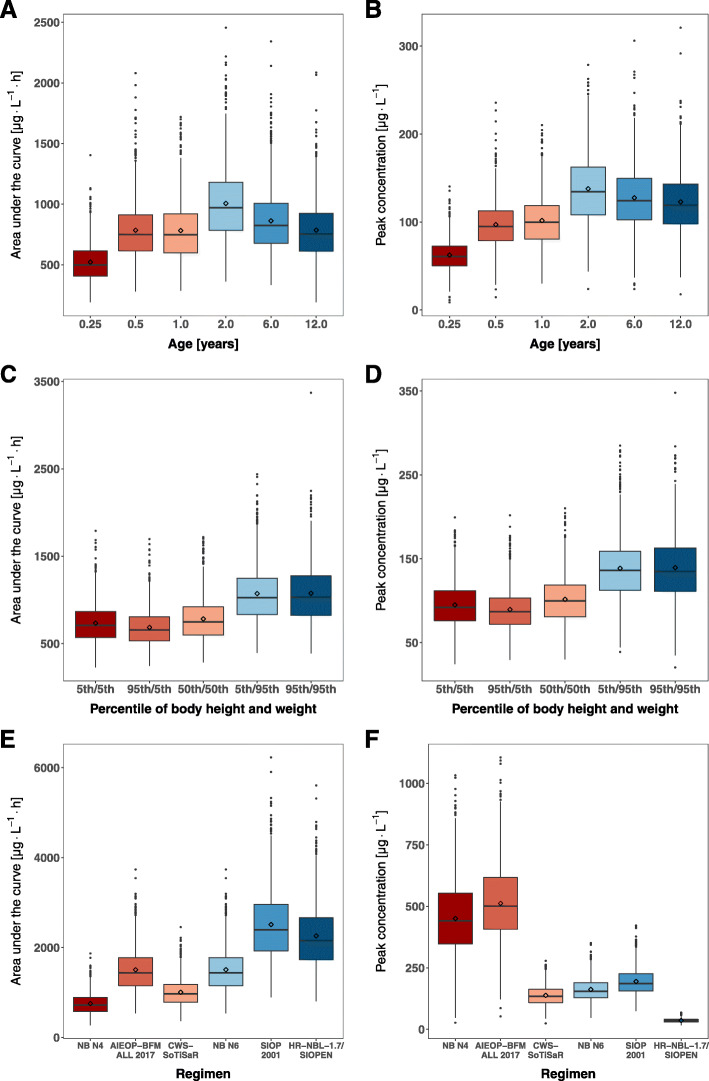
DOX AUC and c_max_ depending on age (**a**, **b**), body composition (**c**, **d**), and treatment regimen (**e**,** f**). For (**a**, **b**) children on the 50th percentile of body height and weight were simulated and for (**c**, **d**) children aged 1 year were simulated. The DOX dose was adopted from the CWS-guidance and doses were reduced in children < 6 months to 67% of the body weight-based dose and in children 6–12 months or < 10 kg to 100% of the body weight-based dose (see Additional file 1). For (**e**, **f**) a median 2-year-old child was simulated (for doses and infusion times see Additional file 1). To display the remaining inter-individual variability that cannot be attributed to the influence of age or body surface area simulations were replicated 1000 times

As simulations of typical AUC and c_max_ values simplifies the real-life situation with large inter-individual variability of DOX PK, taking this variability into account gives us a more genuine impression of variability in AUC and c_max_ in children. It becomes evident that, irrespective of schedule- and age-dependent variations, a substantially broad distribution of individual AUC and c_max_ has to be considered due to the high variability in PK that cannot be sufficiently explained by age and BSA (Fig. 2a, b). For a 2-year-old child treated according to the CWS-guidance, the 5th percentile of simulated AUC values is 565 μg·L^− 1^·h and the 95th percentile is 1588 μg·L^− 1^·h. Corresponding percentiles for simulated c_max_ values are 83 μg·L^− 1^ and 207 μg·L^− 1^.

As a result of conversion rules from BSA-based dosing to body weight-based dosing, therapy intensity will further differ among infants of the same age but heterogeneous in body composition depending on the specific regimen-defined boundaries. For example, simulated median doxorubicin AUC and c_max_ differ more than 30% between a one-year old child on the 95th percentile of body weight who already receives the full BSA-based dose according to the CWS-guidance and a child on the 5th or 50th percentile of body weight who still receives the body-weight-based dose (Fig. 2 c, d).

A comparison of simulated AUC and c_max_ following a single drug administration between selected treatment regimens is displayed in Fig. 2 (e, f). Besides the dose, the duration of infusion determines peak concentrations leading to large differences between treatment regimens. Though the dose is lower in the NB 2016 N4 regimen (15 mg·m^− 2^) compared to the CWS-guidance (20 mg·m^− 2^) median peak concentrations simulated for a 2-year-old child are more than 3 times higher due to the difference in infusion time (30 min vs. 3 h).

### Can we make it better?

**Fig. 3:**
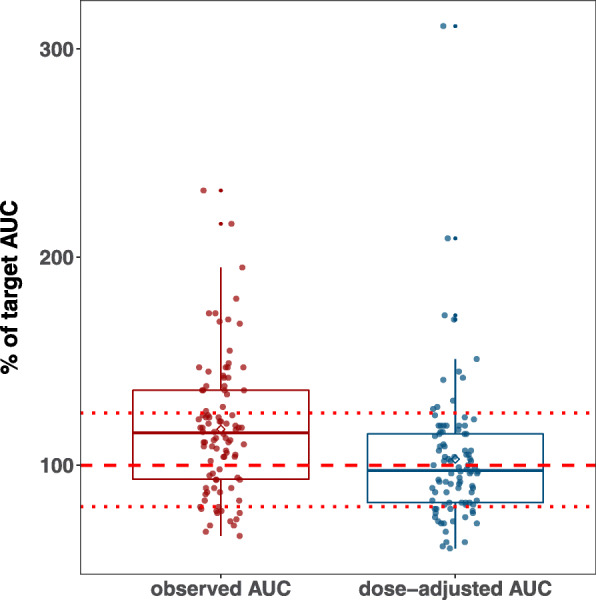
Comparison of observed AUC from 94 patients from the EPOC-MS-001-Doxo trial and dose-adjusted AUC. Adjusted DOX doses were derived from a model-based dose calculation rule. AUC values were calculated based on the *post-hoc* clearance estimates taken from the NONMEM analysis and normalised to the target AUC of a typical 18-year-old boy. The dashed red line indicates the target AUC of 100% and dotted red lines indicate a range of 80–125%

A standardized approach to modify the DOX dose in young children can be derived from the population PK model for DOX. A dosing method that aims to achieve more uniform AUC levels across the age range has been described by our group before [16]. In the present study, we assessed the impact of the suggested dosing method on drug exposure for the 94 children of the EPOC patient population (Fig. 3). Application of this dosing algorithm allows to achieve a defined target AUC without relevant bias (− 2.5%, 95% confidence interval -8–3%), however, variability in drug exposure is still substantial underlined by the small decrease in precision between observed (21%, 95% confidence interval 18–23%) and hypothetical, dose-adjusted AUC values (17%, 95% confidence interval 13–19%) (*p* < 0.05). The percentage of AUC attaining the range of 80–125% around the target AUC was 58.5% for the observed AUC and 69.1% for dose-adjusted AUC values. This difference was not statistically noticeable.

### Defining a common consensus for DOX dosing concepts in children

To strengthen the rationale for dose modifications in young children we conducted a Delphi consensus procedure in which 11 expert paediatric oncologists participated (see Additional file 2 for the 2nd round questionnaire). Those experts represented large paediatric study groups as well as clinical centres.

Two main conclusions can be drawn from this consensus procedure. First, standardisation of dose modifications in young children should be based on the aim of uniform drug exposure across the age range. Secondly, peak levels should be additionally reduced in the youngest patients. Therefore, treatment strategies for children should adapt both the dose and the duration of infusion. As a further conclusion, future DOX-containing regimens for children should be designed such that extremes in therapy intensity are avoided which encourages attempts to further study the concentration-response-relationships of DOX in order to select the most appropriate doses and schedules. A detailed description of the results of the Delphi procedure is presented in Additional file 4.

## Discussion

Among childhood cancer survivors, cardiac disease is the leading non-malignant cause for morbidity and mortality [24]. As the vast majority of children diagnosed with cancer are currently cured [25], the prevention of treatment-related toxicities plays a key role. For DOX and other anthracyclines the relationship of PK measures (e.g. AUC, c_max_) and treatment outcome has not been definitively established. Nevertheless, the reduction of variability in treatment intensity holds promise to better balance tumour efficacy and the risk of toxicity, in particular late cardiac effects. Seemingly arbitrary thresholds for dose modification and conversion rules as part of empirically-derived treatment regimens along with age-dependent differences in individual PK substantially contribute to this variability. Protocol optimisation is needed and might offer the possibility to increase the safety of DOX administration.

A population PK model-based strategy to adapt DOX doses in young children has been described by our group before [16]. A weak point of this method, however, is the lack of evidence for the target PK parameter. Adapting the DOX dose with the aim to achieve more uniform drug exposure across the age range considers that AUC is presumably the most relevant determinant for therapy efficacy [26]. It neglects, however, the higher organ toxicity of DOX in young children and the role of the peak concentration for cardiac toxicity [3, 8–10]. In a situation when definite clinical evidence is lacking a Delphi consensus procedure may help to sharpen the rationale and pharmacological goals of DOX dose modifications in children with cancer. In contrast to open group discussions this approach permits collecting individual opinions and transforming opinions into a group consensus without being influenced by a single opinion leader [27]. Though evidence is scarce, the Delphi procedure allowed clarifying the pharmacological goals of dose modifications and formulating a standardised dosing concept, based on the collective knowledge and opinion of clinical experts. It should be clearly stated that collective opinion must not be erroneously confused with scientific evidence and should not be seen as indisputable fact. A Delphi procedure does not create new knowledge but rather seeks to make optimal use of already existing knowledge [27].

However, with the consented a priori dose adaptation a consistent strategy applicable to all treatment regimens has become available. The Delphi panel confirmed the initially proposed concept which individualises absolute DOX doses based on patient characteristics (age and BSA) that are predictive for DOX PK. Aiming at uniform drug exposure among children treated according to the same protocol thereby appears to be most reasonable as systemic drug exposure has been widely used as a surrogate marker for dose adaptations [28]. An appropriate dosing equation is available based on the population PK model for DOX (cf. formulas 1 & 2 in the “Methods” section) [16]. In this way optimised treatment regimens may allow for a rational choice of the DOX dose in paediatrics, ideally improving the safety of DOX application. As an extension of the dosing concept described in [16], the experts also recommended an additional reduction of peak levels in very young children by prolonged infusion, thus taking into account the presumed influence of peak levels on cardiotoxicity and the higher cardiac risk of very young patients. In conclusion, modifications of treatment strategies in young children should therefore be based on two aspects, adjustment of the dose and of the infusion duration.

As a prerequisite for the proposed dosing concept the target AUC that should serve as reference needs to be specified. In our example we used the AUC expected for a ‘standard’ 18-year-old boy (i.e. an adult patient) as a reference (Fig. 3), as this seems to be straightforward. However, other targets might be even more appropriate. For instance, a target AUC based on the median clearance of a representative patient population has been used for renal function-based carboplatin dosing [29]. Apart from that, the consented prolongation of infusion time in younger children as a measure to reduce peak concentrations might be opposed by clinical practicability (i.e. practicability in an ambulatory care setting) and patient convenience. In addition, the exact influence of infusion time on peak concentrations also requires further investigation.

Constraining the range of DOX doses and infusion times that are applied in current protocols may offer an opportunity to prevent extreme AUC values and, maybe more important, peak concentrations. As described above, a plethora of studies investigated the potentially beneficial impact of prolonged infusion (i.e. lower peak concentration) on cardiac outcome [8–11, 14]. Based on a systematic review of the existing literature, Loeffen and colleagues recommended a DOX infusion duration of at least 1 h in paediatric cancer patients [30]. However, this conclusion does not take into account the administered dose and its impact on c_max_. Additionally, some evidence is available pointing to an increased risk of heart failure with a higher maximal anthracycline dose within 1 week [31]. The avoidance of very short infusion times on the one hand or very high DOX doses on the other hand thus represents a potential measure to reduce the risk of long-term cardiac side effects. This has been unanimously consented by the expert panel but some disagreement arose from the question whether target ranges could be uniformly defined across different tumour types. In contrast to the large variety in DOX administration, there is no data that clearly demonstrate that different tumour entities indeed need specific peak concentrations or drug exposure. Yet, in multi-agent combination chemotherapy regimens adequate DOX therapy intensity will be influenced by the particular combination of chemotherapeutic drugs. Obviously, more research on the dose-concentration-effect relationships in different tumour types is needed to support the establishment of pharmacologically meaningful thresholds and the selection of the most appropriate doses and schedules.

The approach presented herein underlines the value of population PK modelling for treatment optimisation. The DOX population PK model was used to illustrate the complex interplay of dose modifications and PK relationships. Moreover, it provides an opportunity to translate the consented dosing goals into alternative dosing algorithms. It has to be mentioned that the validity of the model-based approach is limited by the small number of patients below the age of 1 year recruited in the EPOC-MS-001-Doxo trial (*N* = 4) with the youngest child being 2.5 months old. Thus, uncertainties of model-based predictions are highest in this age group. Similar is true for highly obese paediatric patients. For a routine use of any model-based dosing recommendation two requirements are thus mandatory. Firstly, it is necessary to further validate the population PK model by assessing its predictive performance in a new patient population which should include relevant numbers of infants and young children [32]. Secondly, the consented dosing concept needs to be validated in a prospectively-designed clinical trial assessing its suitability to target a predefined drug exposure.

One may criticize that the Delphi expert panel was rather small to draw meaningful conclusions. However, standards for panel sizes have not yet been established and in the past, Delphi studies have been performed with virtually any panel size. With similar trained experts a small expert panel may be used with sufficient confidence [33]. Despite the small number of participants, agreement among the experts was strong with relatively little variation for most of the questions. The obtained consensus reflects the perspectives of both relevant paediatric study groups and clinical centres. Nonetheless, further discussion with clinical experts on the findings and potential implementations is highly welcome.

As suggested by Fig. 3, a relatively small reduction in variability of drug exposure can be expected though individualisation of the DOX dose with respect to age and BSA. Large variability is a long-known characteristic of DOX PK. In adults, substantial inter-patient variations of AUC despite standardisation of the dose based on BSA were observed and differences in dose-normalised peak concentrations of more than 10-fold between children with ALL were reported in a study by Frost et al. [34–36]. Adaptive administration of chemotherapeutics based on plasma concentration measurements could provide an opportunity to further reduce variability in drug exposure. Individual PK parameters can be easily predicted based on a few plasma concentration measurements using a Bayesian forecasting approach [28]. It has been shown before that adaptive dosing of chemotherapeutics can result in a narrower and more accurate exposure range compared with standard BSA-based dosing and can positively impact therapeutic outcome [37, 38]. However, in the past several studies revealed unpredictable differences in individual DOX PK between consecutive administrations [34, 39]. In a study by Hempel et al. in paediatric ALL and non-Hodgkin lymphoma patients intra-individual deviations in peak concentration ranged from 3.5 to 198% [39]. In accordance, population PK analysis of data from the EPOC-MS-001-Doxo trial found high intra-individual variability on the central volume of distribution [15]. Due to the high intra-individual variability Hempel et al. concluded that dose individualisation based on monitoring of peak concentrations will not be feasible. In contrast, in a population PK analysis in adults and children older than 3 years intra-individual variability of DOX clearance accounted only for 13% [40]. As drug elimination might be less affected from intra-individual variability adaptive dosing approaches aiming to better control variability in drug exposure could indeed be promising. In fact, within the Delphi process the expert panel members acknowledged that therapeutic drug monitoring might be beneficial at least for defined paediatric patient populations.

Nevertheless, pre-analytical variability affects the uncertainties of pharmacokinetic models and model-based predictions. Further, the implementation of drug monitoring and adaptive dosing approaches in clinical routine is hampered by considerable technical effort and logistical requirements. The development of miniaturised monitoring tests and their delivery to the point-of-care is crucial to overcome these limitations [41].

## Conclusions

Making use of the collective opinion of clinical experts the pharmacological goals of DOX dose modifications have been specified. The consented a priori dose adaptation provides a consistent alternative to the huge diversity of current dosing recommendations for small children thus offering the chance to improve safety of this potent anticancer drug in the most vulnerable patient population. In perspective, the possibility is given to validate a common dose calculation rule in young children across the different study groups taking into account the specific dose intensity for each tumour entity and allowing for a unique drug exposure across age within children of the same tumour entity. Nevertheless, the translation of any model-based dosing recommendation for DOX into clinical practice requires consideration of several key aspects (Table 1).

**Table 1:** Key aspects that need to be considered for clinical implementation of model-based dosing recommendations

**1**	Development and implementation of miniaturised bedside analytics in order to minimise pre-analytical variability and facilitate drug monitoring
**2**	External validation of pharmacokinetic models and, if appropriate, further refinement in order to assess the predictive power and decrease the uncertainties of model predictions
**3**	Development of optimised limited sampling strategies to keep the burden of blood sampling for children at a minimum
**4**	Clinical validation of model-based dosing recommendations in a prospectively designed clinical trial

## Supplementary information

**Additional file 1: Table S1.** Overview on doxorubicin doses, infusion times and dose modifications in young children for selected treatment regimens.

**Additional file 2: Questionnaire S2.** Questionnaire for the second round of the Delphi procedure.

**Additional file 3: Table S3.** Demographics of the 94 patients from the EPOC-MS-001-Doxo trial.

**Additional file 4.** Delphi Procedure S4. Methodology and results of the Delphi consensus procedure.

## Data Availability

The data that support the findings of this study are available from the corresponding author on reasonable request.

## Abbreviations

ALL: Acute lymphoblastic leukaemia
AUC: Area under the concentration-time curve
BSA: Body surface area
c_max_: Peak concentration
DOX: Doxorubicin
PK: Pharmacokinetics
