# Supplementary material for: Can we optimise doxorubicin treatment regimens for children with cancer? Pharmacokinetic simulations and a Delphi consensus procedure
Source: BMC Pharmacol Toxicol. 2020 May 28;21:37. doi: 10.1186/s40360-020-00417-2 (PMC7254632; doi:10.1186/s40360-020-00417-2)
Supplement: Supplementary file 1 — Additional file 1: Table S1. Overview on doxorubicin doses, infusion times and dose modifications in young children for selected treatment regimens. [file 40360_2020_417_MOESM1_ESM.docx]

**Supporting information table S1**

**Can we optimise doxorubicin treatment regimens for children with cancer? Pharmacokinetic simulations and a Delphi consensus procedure**

Christian Siebel^1^, Gudrun Würthwein^1^, Claudia Lanvers-Kaminsky^1^, Nicolas André^2^, Frank Berthold^3^, Ilaria Castelli^4^, Pascal Chastagner^5^, François Doz^6^, Martin English^7^, Gabriele Escherich^8^, Michael C. Frühwald^9^, Norbert Graf^10^, Andreas Groll^1^, Antonio Ruggiero^11^, Georg Hempel^12^, Joachim Boos^1^

**Correspondence:** Joachim Boos, Department of Paediatric Haematology and Oncology, University Children’s Hospital Muenster, Albert-Schweitzer-Campus 1, A1, 48149 Muenster. E-mail: [boos@ukmuenster.de](mailto:boos@ukmuenster.de); Tel: +49 251 83-55657; Fax: +49 251 83-55740

**Table S1:** Overview on doxorubicin doses, infusion times and dose modifications in young children for selected treatment regimens

| **Protocol** | **Dose and infusion time** | **Dose modification** |  |
| --- | --- | --- | --- |
| NB Registry 2016 | 15 mg·m^-2^, 0.5 h, day 1+3+5 (N4) | < 12 months: | 100 % of body weight-based dose |
|  | 30 mg·m^-2^, 4 h, day 6+7 (N6) | ≥ 12 months and < 10 kg: | 100 % of body weight-based dose (0.5 mg·kg^-1^ (N4); 1.0 mg·kg^-1^ (N6)) |
|  |  | ≥ 12 months and ≥ 10 kg: | 100 % of BSA dose |
|  |  |  |  |
| CWS-SoTiSaR | 2 x 20 mg·m^-2^, 3 h, day 1+2 | < 6 months: | 67 % of body weight-based dose |
|  | (every 8 - 12 h) | ≥ 6 months or ≤ 10 kg: | 100 % of body weight-based dose (0.67 mg·kg^-1^) |
|  |  | ≥ 12 months and > 10 kg: | 100 % of BSA dose |
|  |  |  |  |
| Ewing 2008 | 20 mg·m^-2^, 4 h, day 1+2+3 | - | - |

BSA, body surface area

**Table S1:** Overview on doxorubicin doses, infusion times and dose modifications in young children for selected treatment regimens

| NHL-BFM Registry 2012 | 25 mg·m^-2^, 1 h, day 4+5 (B-NHL/B-AL) | ≤ 6 months: | 67 % of BSA dose |
| --- | --- | --- | --- |
|  | 30 mg·m^-2^, 1h, day 8+15+22+29 (LBL) | > 6 months and < 12 months: | 75 % of BSA dose |
|  |  | ≥ 12 months: | 100 % of BSA dose |
|  |  |  |  |
| AIEOP-BFM ALL 2017 | 30 mg·m^-2^, 1 h, day 8+15+22+29 | < 6 months: | 67 % of BSA dose |
|  |  | ≥ 6 months and < 12 months: | 75 % of BSA dose |
|  |  | ≥ 12 months: | 100 % of BSA dose |
|  |  |  |  |
| CoALL-08-09 | 30 mg·m^-2^, 24 h, day 1+8+22+29 | - | - |
|  |  |  |  |
| HR-NBL-1.7/SIOPEN | 45 mg·m^-2^, 48 h | ≤ 5 kg: | 67 % of body weight-based dose |
|  |  | > 5 kg and < 12 kg: | 100 % of body weight-based dose (1.5 mg·kg^-1^) |
|  |  | ≥ 12 kg | 100 % of BSA dose |
|  |  |  |  |
| SIOP 2001/GPOH | 50 mg·m^-2^, 6 h | < 6 months: | 50 % of BSA dose |
|  |  | ≥ 6 months or < 12 kg: | 67 % of BSA dose |
|  |  | ≥ 6 months and ≥ 12 kg: | 100 % of BSA dose |
|  |  |  |  |
| European Rhabdoid Registry | 75 mg·m^-2^, 48 h | < 6 months: | 100 % of body weight-based dose |
|  |  | ≥ 6 months and < 10 kg | 100 % of body weight-based dose (2.5 mg·kg^-1^) |
|  |  | ≥ 6 months and ≥ 10 kg | 100 % of BSA dose |

BSA, body surface area
