# Supplementary material for: Can we optimise doxorubicin treatment regimens for children with cancer? Pharmacokinetic simulations and a Delphi consensus procedure
Source: BMC Pharmacol Toxicol. 2020 May 28;21:37. doi: 10.1186/s40360-020-00417-2 (PMC7254632; doi:10.1186/s40360-020-00417-2)
Supplement: Supplementary file 2 — Additional file 2: Questionnaire S2. Questionnaire for the second round of the Delphi procedure. [file 40360_2020_417_MOESM2_ESM.pdf]

Delphi survey to establish a consensus on doxorubicin dosing in infants and children

Round 2

Part I: consensus on doxorubicin dose adjustments in infants/younger children

Part I of the questionnaire focuses on differences in the individually experienced doxorubicin therapy intensity (quantified as AUC and cmax) that can be observed within specific treatment protocols. Of particular importance in this context are differences between infants/younger children and older children.

The aims are:

- to clarify the rational for dose adjustments in infants/younger children which are currently part of almost all treatment protocols for paediatric cancers
- to identify pharmacokinetic targets for more rational dose adjustments

Some of the questions in this questionnaire use the CWS-2002/CWSSoTiSaR protocol as an example. Please note that this protocol has been representatively chosen to visualize the effects of doxorubicin dosing on the individually experienced therapy intensity. However, simulations confirmed that other protocols lead to similar results.

Please state whether you agree or disagree with each of the statements listed below (1 = strongly disagree; 5 = strongly agree) or, when appropriate, vote for one of the suggested options:

1) *Age-dependent differences in doxorubicin plasma concentration-time curves as observed within current protocols are of clinical relevance (see fig. 1).*

○	○	○	○	○
strongly disagree	disagree	neutral	agree	strongly agree

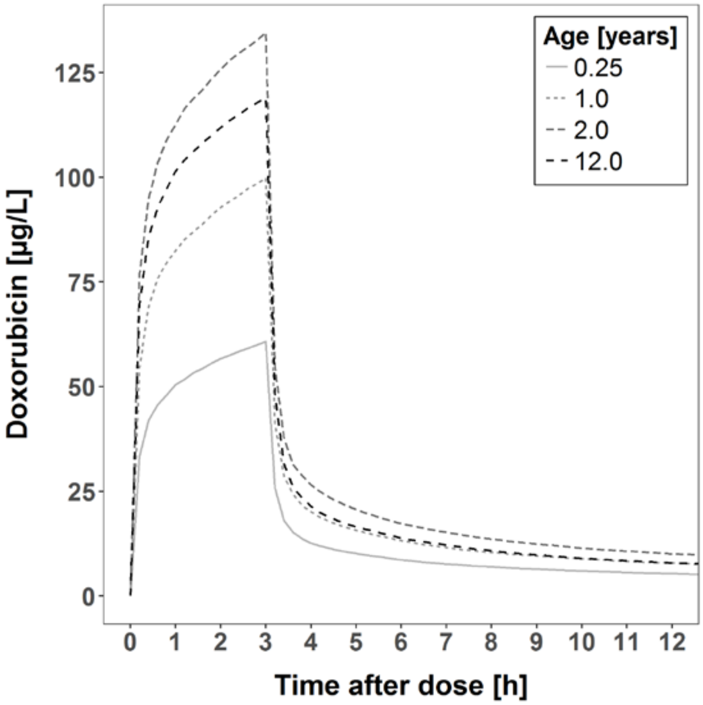

Figure 1: Median plasma concentration-time curves simulated for generic children with median body height and weight and treated according to the CWS-2002/CWSSoTiSaR protocol. Dose was reduced in children below 1 year or weighing less than 10 kg as suggested by the protocol.

2) *Doxorubicin dose should be adjusted in infants/younger children to reduce the risk of cardiac injury.*

○ strongly disagree ○ disagree ○ neutral ○ agree ○ strongly agree

3) *Would you accept a potentially lower tumour efficacy in infants/younger children in favour of a reduced cardiotoxic risk?*

○ strongly disagree ○ disagree ○ neutral ○ agree ○ strongly agree

4) *Doxorubicin dose should be adjusted to compensate individual differences in pharmacokinetics, e.g. a priori dose adjustment to age and BSA to compensate for a reduced clearance in infants/younger children.*

○	○	○	○	○
strongly disagree	disagree	neutral	agree	strongly agree

5) *Doxorubicin dose should be adjusted in infants/younger children to achieve defined target:*

a) AUC
- ○ *uniform across age groups*
- ○ *lower in infants/younger children*
- ○ *not necessary to adjust to AUC*

b) cmax
- ○ *uniform across age groups*
- ○ *lower in infants/younger children*
- ○ *not necessary to adjust to cmax*

6) In figure 2 the change of doxorubicin AUC (A,C) and cmax (B,D) **with age** is simulated for the CWS-2002/CWSSoTiSaR protocol and different alternative dose adjustments.

In a cohort of 101 paediatric cancer patients the EPOC-MS-001 Doxo trial (EudraCT-Nr: 2009-011454-17) confirmed that doxorubicin clearance is lower in younger children compared to older children. As a consequence, the AUC of a 15-month-old child is considerably higher than the AUC of an 18-year-old and steadily declines with age (fig. 2 (A)). Correspondingly, peak plasma levels behave similar (fig. 2 (B)). However, in this protocol children below 1 year or weighing less than 10 kg are subject to dose adjustments which lead to extreme differences in AUC and cmax in these children (fig. 2 (A,B)).

Changing the strategy of dose adjustment allows to tailor the experienced therapy intensity towards specific goals (fig. 2 (C,D)). The alternative dose adjustments applied here were designed such that an equal AUC, a gradual increase or a continuous increase in AUC could be achieved.

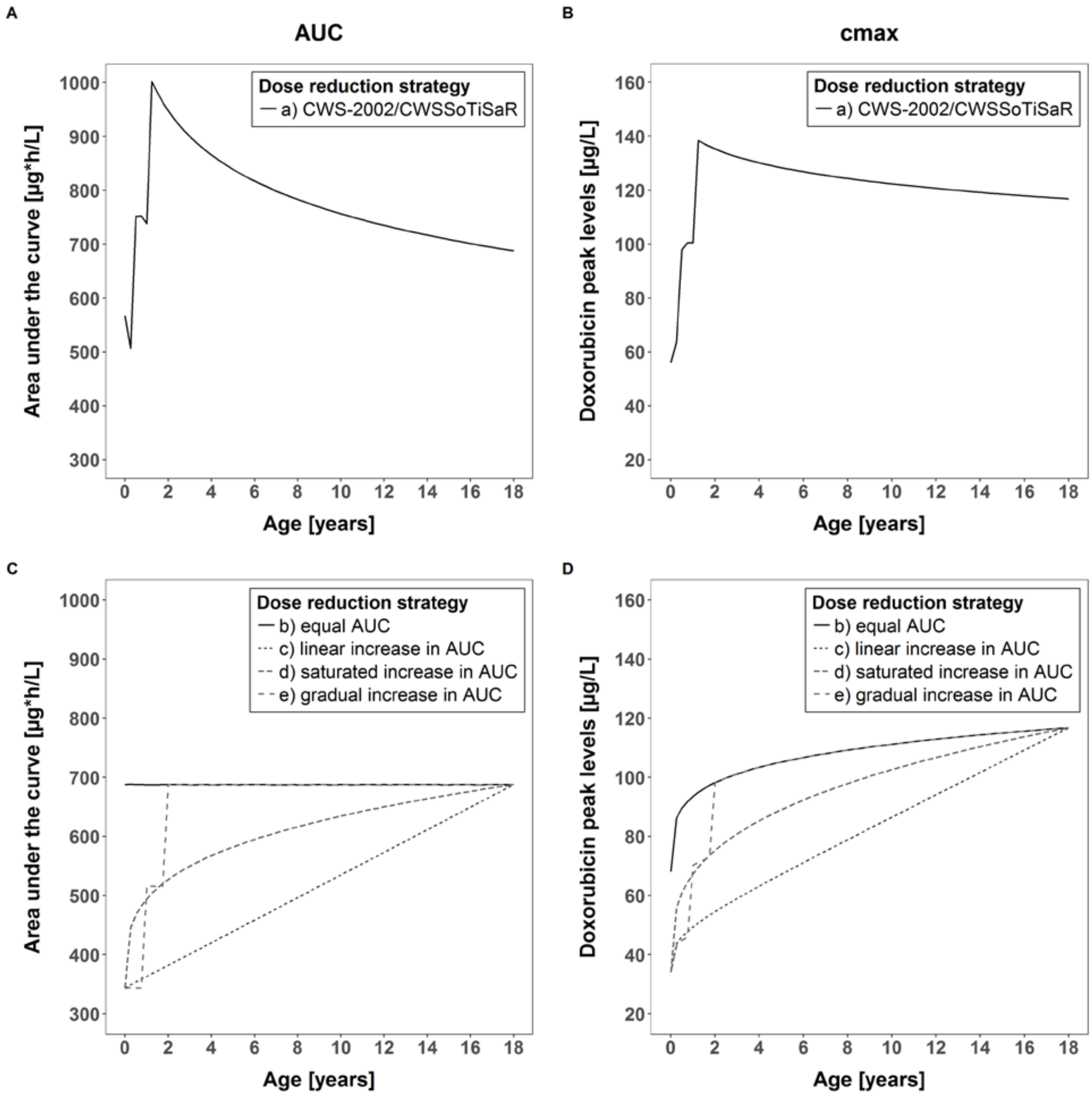

Figure 2: Simulation of median AUC (A,C) and cmax (B,D) for different alternative dose adjustments. Dose was adjusted (a) according to the current CWS-2002/CWSSoTiSaR protocol; (b) to achieve an equal AUC across age; (c) to achieve a continuous, linear increase in AUC with age; (d) to achieve a continuous, saturated increase in AUC with age; (e) to achieve a gradual increase in AUC. (B,D) visualize the effect of the applied dose adjustments on cmax. In general, development of cmax with age follows the AUC curves.

Which of the suggested dose reduction strategies would you prefer?

- ◯ *a) AUC/cmax achieved with dose adjustment currently implemented in the CWS-2002/CWSSoTiSaR protocol*
- ◯ *b) dose adjustment to achieve equal AUC across age*
- ◯ *c) dose adjustment to achieve continuous, linear increase in AUC with age*
- ◯ *d) dose adjustment to achieve continuous, saturated increase in AUC with age*
- ◯ *e) dose adjustment to achieve gradual increase in AUC with age*
- ◯ *f) neither*

7) In figure 3 (A) doxorubicin concentration-time curves were simulated for children aged 0.5 – 6 years. The children were dosed to achieve the same AUC. However, infusion time was prolonged in an age-dependent manner from 3 hours up to 6 hours in order to prevent high peak plasma levels in younger children. Figure 3 (B) illustrates how changing the infusion time translates into peak plasma levels.

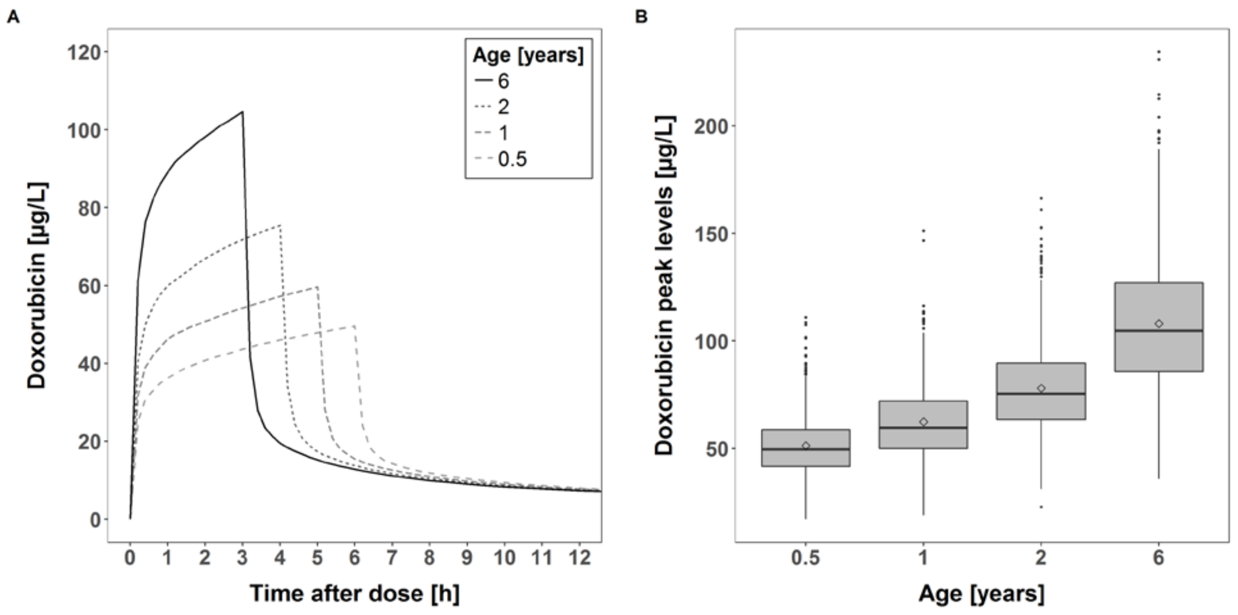

Figure 3: Simulated median doxorubicin concentration-time curves (A) and peak plasma levels (B) for generic children aged 0.5-6 years. Doxorubicin dose was adjusted such that the children achieve an equal AUC. Infusion time was set from 3 hours in a 6-year-old child to 6 hours in a 0.5-year-old child to reduce the peak plasma levels in these children. (B) The simulation was performed in 1000 replicates to visualize variability.

*Would you accept **age-specific** adjustment of infusion time within a given protocol, e.g. prolonged infusion in infants compared to older children to achieve lower cmax?*

○	○	○	○	○
strongly disagree	disagree	neutral	agree	strongly agree

8) *Doxorubicin dose should be adjusted to body composition to achieve uniform AUC and cmax in children of the same age.*

○	○	○	○	○
strongly disagree	disagree	neutral	agree	strongly agree

Part II: Definition of common targets to guide doxorubicin administration

Part II of the questionnaire focuses on differences between current treatment protocols. In principle, one can distinguish between protocols with short infusion time that lead to plasma concentration-time curves with high peak levels and those protocols that can be characterized by significantly lower peak levels but prolonged exposition. Furthermore, high variability in single doxorubicin doses and consequently in AUC exists between current protocols (see fig. 4).

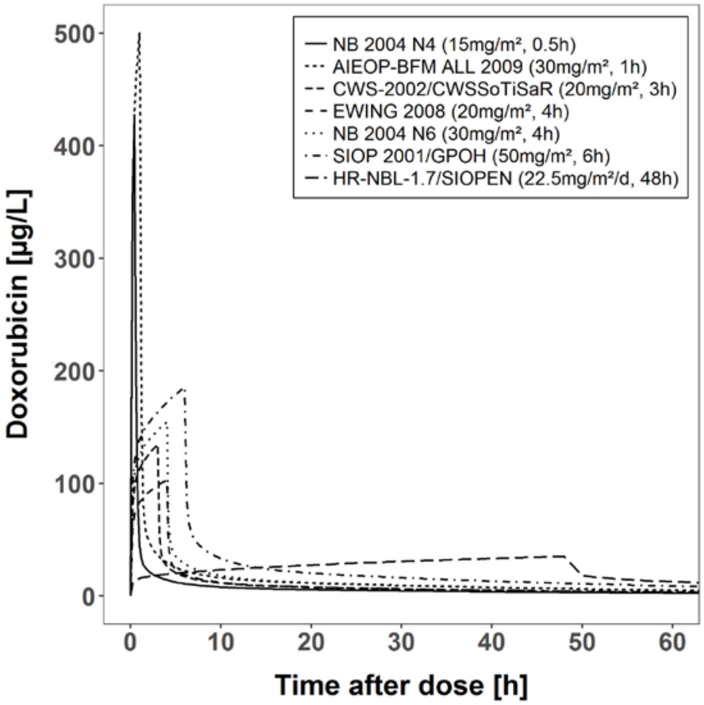

Figure 4: Median doxorubicin plasma concentration-time curves simulated for a generic 2-year-old child and treated according to different currently used treatment protocols.

In the following, various aspects of doxorubicin administration are questioned.

The aim is:

- to identify common targets between current treatment protocols that might guide doxorubicin administration

Please state whether you agree/disagree with each aspect.

*9) To reduce the risk of **cardiotoxic side effects** it is desirable to establish:*

	1<br>strongly disagree	2<br>disagree	3<br>neutral	4<br>agree	5<br>strongly agree
a) Maximum-allowed AUC	○	○	○	○	○
b) Maximum-allowed cmax	○	○	○	○	○
c) Maximum-allowed time over threshold	○	○	○	○	○

*10) To guarantee **appropriate tumour efficacy** it is desirable to establish:*

	1<br>strongly disagree	2<br>disagree	3<br>neutral	4<br>agree	5<br>strongly agree
a) Minimum-needed AUC	○	○	○	○	○
b) Minimum-needed cmax	○	○	○	○	○
c) Minimum needed time over threshold	○	○	○	○	○

*11) Establish **uniform targets across different tumour entities/treatment protocols**:*

	1<br>strongly disagree	2<br>disagree	3<br>neutral	4<br>agree	5<br>strongly agree
a) Uniform target AUC	○	○	○	○	○
b) Uniform target cmax	○	○	○	○	○
c) Uniform target time over threshold	○	○	○	○	○

12) *Therapeutic drug monitoring of doxorubicin could provide additional benefit for defined patient populations.*

○	○	○	○	○
strongly disagree	disagree	neutral	agree	strongly agree

Part III: Additional aspects

This part refers to additional aspects that could potentially influence doxorubicin administration. Please state whether you consider the following aspects as relevant or not.

13) *Relevance of adapting doxorubicin dosing/target levels to special patient populations:*

	1 not relevant	2 slightly relevant	3 moderately relevant	4 relevant	5 highly relevant
Infants/children with good prognosis disease	○	○	○	○	○
Tumour predisposition syndromes	○	○	○	○	○
Down syndrome patients with AML/ALL	○	○	○	○	○
Syndromes with higher toxicity of chemotherapy (e.g. Fanconi anaemia)	○	○	○	○	○
Other:					

14) *Relevance of further individual patient characteristics that might be considered for guiding the doxorubicin dose:*

	1<br>not relevant	2<br>slightly relevant	3<br>moderately relevant	4<br>relevant	5<br>highly relevant
Use of cardioprotectant	○	○	○	○	○
Other comedication	○	○	○	○	○
Mediastinal/lung radiotherapy	○	○	○	○	○
Pharmacogenetics analysis	○	○	○	○	○
Suggested genetic markers?					
Use of liposomal doxorubicin	○	○	○	○	○
Other:					
