# Supplementary material for: Can we optimise doxorubicin treatment regimens for children with cancer? Pharmacokinetic simulations and a Delphi consensus procedure
Source: BMC Pharmacol Toxicol. 2020 May 28;21:37. doi: 10.1186/s40360-020-00417-2 (PMC7254632; doi:10.1186/s40360-020-00417-2)
Supplement: Supplementary file 3 — Additional file 3: Table S3. Demographics of the 94 patients from the EPOC-MS-001-Doxo trial. [file 40360_2020_417_MOESM3_ESM.docx]

**Supporting information table S3**

**Can we optimise doxorubicin treatment regimens for children with cancer? Pharmacokinetic simulations and a Delphi consensus procedure**

Christian Siebel^1^, Gudrun Würthwein^1^, Claudia Lanvers-Kaminsky^1^, Nicolas André^2^, Frank Berthold^3^, Ilaria Castelli^4^, Pascal Chastagner^5^, François Doz^6^, Martin English^7^, Gabriele Escherich^8^, Michael C. Frühwald^9^, Norbert Graf^10^, Andreas Groll^1^, Antonio Ruggiero^11^, Georg Hempel^12^, Joachim Boos^1^

**Correspondence:** Joachim Boos, Department of Paediatric Haematology and Oncology, University Children’s Hospital Muenster, Albert-Schweitzer-Campus 1, A1, 48149 Muenster. E-mail: [boos@ukmuenster.de](mailto:boos@ukmuenster.de); Tel: +49 251 83-55657; Fax: +49 251 83-55740

**Table S3:** Demographics of the 94 patients from the EPOC-MS-001-Doxo trial

|  | **Median** | **Range** |
| --- | --- | --- |
| **Age [years]** | 5.32 | 0.2 - 17.7 |
| **Height [cm]** | 111 | 52 - 194 |
| **Weight [kg]** | 19.3 | 3.6 - 88.1 |
| **Body surface area [m²]** | 0.77 | 0.23 - 2.05 |
| **Administered dose [mg]^a^** | 25.0 | 2.4 - 57.0 |
| **Infusion duration [h]** | 3.92 | 0.25 - 24.0 |

^a^ Refers to a single doxorubicin administration
