# Supplementary material for: Can we optimise doxorubicin treatment regimens for children with cancer? Pharmacokinetic simulations and a Delphi consensus procedure
Source: BMC Pharmacol Toxicol. 2020 May 28;21:37. doi: 10.1186/s40360-020-00417-2 (PMC7254632; doi:10.1186/s40360-020-00417-2)
Supplement: Supplementary file 4 — Additional file 4. Delphi Procedure S4. Methodology and results of the Delphi consensus procedure. [file 40360_2020_417_MOESM4_ESM.docx]

**Supporting information Delphi procedure S4**

**Can we optimise doxorubicin treatment regimens for children with cancer? Pharmacokinetic simulations and a Delphi consensus procedure**

Christian Siebel^1^, Gudrun Würthwein^1^, Claudia Lanvers-Kaminsky^1^, Nicolas André^2^, Frank Berthold^3^, Ilaria Castelli^4^, Pascal Chastagner^5^, François Doz^6^, Martin English^7^, Gabriele Escherich^8^, Michael C. Frühwald^9^, Norbert Graf^10^, Andreas Groll^1^, Antonio Ruggiero^11^, Georg Hempel^12^, Joachim Boos^1^

**Correspondence:** Joachim Boos, Department of Paediatric Haematology and Oncology, University Children’s Hospital Muenster, Albert-Schweitzer-Campus 1, A1, 48149 Muenster. E-mail: [boos@ukmuenster.de](mailto:boos@ukmuenster.de); Tel: +49 251 83-55657; Fax: +49 251 83-55740

**Methodology and results of the Delphi consensus procedure**

**Methodology**

Design of questionnaires and conduct of survey

The aim of a Delphi survey has been described to achieve consensus within a group of experts in an area with high uncertainty or a lack of empirical evidence. As a key feature, a Delphi survey is designed as an iterative process based on a series of questionnaires and informed by a feedback of the groups’ responses which should encourage participants to reassess and, where appropriate, to change their opinions [1, 2]. The Delphi procedure consisted of three rounds. In a first qualitative pilot round participants were asked to answer a set of open-ended questions regarding pharmacological goals of DOX administration. The aim of this first round was to provide participants with a summary of relevant background information involving PK simulations and a description of current DOX administration schedules and to support the development of the definitive set of questions forming the 2nd and 3rd round questionnaires. In these two rounds more specific questions had to be answered by rating for agreement on a 5-point Likert scale (1 = strongly disagree; 5 = strongly agree) or for relevance (1 = not relevant; 5 = highly relevant), respectively. The 2nd and 3rd round questionnaires were structured in three sections covering (I) dose modifications in infants/young children, (II) general goals of DOX administration and (III) additional aspects that have been mentioned by the participants in the 1st round (see additional file 2 for the 2nd round questionnaire). Following round two responses were summarized and fed back to the participants within the third questionnaire. In view of the groups’ responses participants were asked to reassess their initial judgement and to re-rate all questions. Answering the questions was expected to take approx. 30 min for the 1st and approx. 15 min for the 2nd and 3rd round questionnaire. Three weeks after the initial distribution of each questionnaire a first reminder was sent and a subsequent reminder was sent two weeks later. Questionnaires and reminders were sent as emails. During the whole Delphi procedure, participants remained anonymous among each other. To maintain anonymity emails to the whole panel were sent as blind copies.

Selection of panellists

Overall, 28 experts in paediatric oncology from clinical centres in four European countries (France, Italy, Germany, United Kingdom) were invited to participate in this Delphi procedure. Invited experts were either EPOC consortium partners (20 experts) or representatives of linked clinical trials (8 experts). An invitation letter was sent to all panellists by email. Upon confirmation of participation the first round questionnaire was sent.

Analysis

Qualitative content analysis was used to summarise responses from the first round questionnaire [3]. Responses were paraphrased and generalized to a first set of categories. Categories were then further reduced and summarized where appropriate. By coding all responses categories were refined. To control the adequacy of the final coding system this was repeated by a second group member. In case of disagreement the reason for disagreement was discussed and the coding system adjusted if necessary. Categories were then used to develop the definitive set of questions. Ratings from round two and three were summarized by specifying range and median. For each question the level of agreement was calculated as the combined percentage of ratings 4 and 5 on the 5-point Likert scale. If questions had not to be answered on a Likert scale (questions 5 & 6 in table 1) percentage of each individual answer was calculated. A level of agreement above 67 % was a priori considered as consensus.

**Results**

The Delphi consensus procedure was conducted between September 2017 and April 2018. Overall, 28 experts were invited to participate in the Delphi procedure of whom 11 agreed to participate, one expert refused and 16 did not respond. Though 11 experts initially agreed to participate only 8 completed the first pilot phase questionnaire. The 2nd and 3rd round questionnaires were each completed by 11 experts. Both clinical centres (7 experts) and relevant paediatric study groups (4 experts) were represented in the final Delphi panel. The median score, range of scores and the level of agreement for the 2nd and 3rd Delphi round are summarised in tables 1 – 3.

Standardising dose modifications in infants/young children

Panel members generally acknowledged the clinical relevance of individual differences in systemic therapy intensity which arise from regimen-specific dose modifications as well as individual differences in PK. Nevertheless, dose modifications are still considered necessary to reduce the risk of cardiac injury in the very young. To improve the current practice, experts agreed on a standardised a priori dose adaptation that takes into account patient’s age and BSA thus allows compensating for age-dependent differences in PK. The pharmacological goal of targeting equal AUC levels across the age range was deemed appropriate. Further, a reduction of peak concentrations in younger children was favoured, albeit only 8 out of 11 experts agreed. This could be achieved by a prolongation of infusion time in these children. As current conversion rules from BSA- to body weight-based dosing can be expected to cause arbitrary differences in therapy intensity among children of the same age this should be avoided by future dose adaptations. Any reduction of the cardiotoxic risk in the high-risk population of very young children at the expense of a potentially lower tumour efficacy was not accepted by the majority of the panel members (table 1).

**Table 1:** Results of the Delphi consensus procedure (part 1).

| **Question** | | | **Median**^a^ | | | | **Range**^a^ | | **Level of agreement [%]**^b^ | |
| --- | --- | --- | --- | --- | --- | --- | --- | --- | --- | --- |
|  |  | Round 2 | | | 3 | | 2 | 3 | 2 | 3 |
| **1** | **clinical relevance** of age-dependent differences in plasma concentration-time curves | | 5 | | | 5 | 3-5 | 4-5 | 82 | **100** |
| **2** | dose adjustment in infants to **reduce the risk of cardiac injury** | | 4 | | | 4 | 2-5 | 3-5 | 82 | **91** |
| **3** | **accept potentially lower tumour efficacy** in favour of a reduced cardio-toxic risk | | 3 | | | 3 | 2-5 | 2-5 | 27 | 9 |
| **4** | **a priori dose adjustment to age and BSA** to compensate individual differences in PK | | 4 | | | 4 | 4-5 | 4-5 | 100 | **100** |
|  |  | |  | | |  |  |  |  |  |
| **5** | adjust dose in infants/younger children to **achieve defined target**: | |  | | |  |  |  |  |  |
|  | 1. AUC | | uniform across age groups | | | | | | 73 | **100** |
|  |  | | lower in infants/younger children | | | | | | 18 | 0 |
|  |  | | not necessary to adjust to AUC | | | | | | 0 | 0 |
|  | 1. cmax | | uniform across age groups | | | | | | 18 | 18 |
|  |  | | lower in infants/younger children | | | | | | 64 | **73** |
|  |  | | not necessary to adjust to cmax | | | | | | 18 | 9 |
| **6** | **further specification** of alternative dose reduction strategies^c^ | | equal AUC across age (option b) | | | | | | 73 | **91** |
|  |  | |  |  | | |  |  |  |  |
| **7** | accept **age-specific adjustment of infusion time** within a given protocol to reduce cmax | | 4 | 4 | | | 2-5 | 2-5 | 82 | **82** |
| **8** | **adjust dose to body composition** to achieve uniform AUC/cmax in children of the same age | | 4 | 4 | | | 2-5 | 4-5 | 82 | **100** |

PK, pharmacokinetic; AUC, area under the concentration-time curve; cmax, peak concentration

^a^ On a 5-point Likert scale (1 = strongly disagree; 5 = strongly agree)

^b^ Combined percentage of ratings 4 and 5 on the 5-point Likert scale

^c^ For question 6 only the most selected option is shown

Evaluation of general goals of DOX administration

Chemotherapy regimens for childhood malignancies vary substantially in dose and infusion time with a large impact on systemic therapy intensity. Given the unclear role of PK characteristics such as AUC and cmax we asked whether it might be desirable to target certain minimum and maximum therapy intensity thresholds, meaning in practice to constrain the range of currently applied doses and infusion times. The participating experts considered AUC, cmax as well as the time of exposure to be important and favoured the establishment of both minimum and maximum threshold levels to balance the risk of cardiac side effects and tumour efficacy. We further asked whether such target ranges should be uniformly defined across different tumour entities. Here, outcome is not unequivocal as levels of agreement ranged from 64 % (7/11 ratings) to 73 % (8/11 ratings). Apart from a priori dose adaptations, all experts agreed that defined patient populations might additionally benefit from therapeutic drug monitoring approaches for DOX (table 2).

**Table 2:** Results of the Delphi consensus procedure (part 2).

| **Question** | | | **Median**^a^ | | **Range**^a^ | | **Level of agreement [%]**^b^ | |
| --- | --- | --- | --- | --- | --- | --- | --- | --- |
|  |  | Round 2 | | 3 | 2 | 3 | 2 | 3 |
| **9** | establish **maximum-allowed PK targets** to reduce the risk of cardiotoxic side effects | |  |  |  |  |  |  |
|  | 1. maximum-allowed AUC | | 4 | 4 | 4-5 | 4-5 | 100 | **100** |
|  | 1. maximum-allowed cmax | | 4 | 4 | 4-5 | 4-5 | 100 | **100** |
|  | 1. maximum-allowed time over threshold | | 4 | 4 | 2-5 | 4-5 | 73 | **100** |
| **10** | establish **minimum-allowed PK targets** to guarantee appropriate tumour efficacy | |  |  |  |  |  |  |
|  | 1. minimum-needed AUC | | 4 | 4 | 1-5 | 4-5 | 91 | **100** |
|  | 1. minimum-needed cmax | | 4 | 4 | 2-5 | 4-5 | 82 | **100** |
|  | 1. minimum needed time over threshold | | 4 | 4 | 3-5 | 4-5 | 91 | **100** |
| **11** | establish **uniform targets across different tumour entities/treatment protocols** | |  |  |  |  |  |  |
|  | 1. uniform target AUC | | 4 | 4 | 2-5 | 2-4 | 64 | **73** |
|  | 1. uniform target cmax | | 3 | 4 | 2-5 | 2-4 | 45 | 64 |
|  | 1. uniform target time over threshold | | 4 | 4 | 2-5 | 2-4 | 55 | **73** |
| **12** | **therapeutic drug monitoring** of doxorubicin to provide additional benefit for defined patient populations | | 5 | 5 | 4-5 | 4-5 | 100 | **100** |

PK, pharmacokinetic; AUC, area under the concentration-time curve; cmax, peak concentration

^a^ On a 5-point Likert scale (1 = strongly disagree; 5 = strongly agree)

^b^ Combined percentage of ratings 4 and 5 on the 5-point Likert scale

Further aspects

In their answers to the open-ended questions of the 1st Delphi round panel members raised additional aspects which were addressed in a separate section of the 2nd and 3rd round questionnaires. Adjusting DOX administration to the particular clinical needs of special patient populations was found to be relevant, which where infants/children with good prognosis disease, patients with tumour predisposition syndromes, Down syndrome patients with AML/ALL, and syndromes associated with higher toxicity of chemotherapy (e.g. Fanconi anaemia). Mediastinal/lung radiotherapy, pharmacogenetic analysis and use of liposomal DOX were considered as potentially relevant factors for DOX administration. Other co-medication and the use of a cardioprotectant were not regarded relevant (table 3).

**Table 3:** Results of the Delphi consensus procedure (part 3).

| **Question** | | | **Median**^a^ | | | **Range**^a^ | | **Level of agreement [%]**^b^ | |
| --- | --- | --- | --- | --- | --- | --- | --- | --- | --- |
|  |  | Round 2 | | | 3 | 2 | 3 | 2 | 3 |
| **13** | relevance of adapting doxorubicin dosing to **special patient populations**^c^ | | |  |  |  |  |  |  |
|  | - infants/children with good prognosis disease | | | 4 | 4 | 4-5 | 4-5 | 100 | **100** |
|  | - tumour predisposition syndromes | | | 4 | 4 | 1-5 | 1-5 | 73 | **73** |
|  | - Down syndrome patients with AML/ALL | | | 5 | 5 | 4-5 | 4-5 | 100 | **100** |
|  | - syndromes with higher toxicity of chemotherapy | | | 5 | 5 | 4-5 | 4-5 | 100 | **100** |
| **14** | relevance of further **individual patient characteristics** for doxorubicin dosing | | |  |  |  |  |  |  |
|  | - use of cardioprotectant | | | 3 | 3 | 1-5 | 1-5 | 45 | 36 |
|  | - other co-medication | | | 4 | 4 | 1-5 | 2-4 | 55 | 64 |
|  | - mediastinal/lung radiotherapy | | | 5 | 5 | 2-5 | 2-5 | 91 | **91** |
|  | - pharmacogenetic analysis^d^ | | | 4 | 4 | 2-5 | 2-5 | 82 | **91** |
|  | - use of liposomal doxorubicin | | | 4 | 4 | 2-5 | 2-5 | 82 | **82** |

PK, pharmacokinetic; AUC, area under the concentration-time curve; cmax, peak concentration

^a^ On a 5-point Likert scale (1 = not relevant; 5 = highly relevant)

^b^ Combined percentage of ratings 4 and 5 on the 5-point Likert scale

^c^ Additional patient populations have been suggested by the participants during round two: neuroblastoma patients (low/intermediate vs. high risk), frail patients and obese patients

^d^ Suggested pharmacogenetics markers include polymorphisms in genes encoding for carbonyl reductases, ABC transporters, catalase, nitric oxide synthase, solute carrier transport proteins, and superoxide dismutase

References

1. Hasson F, Keeney S, McKenna H. Research guidelines for the Delphi survey technique. Journal of Advanced Nursing. 2000;32:1008–15. doi:10.1046/j.1365-2648.2000.t01-1-01567.x.

2. Powell C. The Delphi technique: myths and realities. Journal of Advanced Nursing. 2003;41:376–82.

3. Mayring P. Qualitative Inhaltsanalyse: Grundlagen und Techniken. Weinheim: Beltz Verlagsgruppe; 2010.
